# Two Diagnostic Challenges in Hepatitis B Serology: Low HBsAg S/CO Values and Isolated Anti-HBc Positivity

**DOI:** 10.3390/pathogens15070777

**Published:** 2026-07-22

**Authors:** Şerife Yılmaz Gürbüz, Oğuzhan Yağdı, Erhan Başar

**Affiliations:** 1 Department of Medical Microbiology, Medical Faculty, Karabuk University, 78050 Karabuk, Turkey; 2 Medical Microbiology Laboratory, Karabuk Training and Research Hospital, 78100 Karabuk, Turkey; oguzhanyagdi@gmail.com (O.Y.); drerhanbasar@hotmail.com (E.B.); 3 Medical Microbiology Laboratory, Çorlu State Hospital, 59850 Tekirdağ, Turkey

**Keywords:** Hepatitis B virus, HBsAg, S/CO ratio, isolated anti-HBc, HBV DNA, occult hepatitis B, ROC analysis

## Abstract

Hepatitis B surface antigen (HBsAg) signal-to-cutoff (S/CO) values and isolated anti-HBc positivity represent two significant diagnostic challenges in hepatitis B virus (HBV) serology. This retrospective study, conducted in Karabuk between 2021 and 2025, aimed to evaluate the correlation between these profiles and HBV DNA positivity to determine the optimal S/CO thresholds for confirmatory testing and clarify the clinical relevance of isolated anti-HBc reactivity. Among 17,356 patients screened for anti-HBc, isolated anti-HBc positivity was identified in 506 patients (2.9%), of whom 3.2% had detectable HBV DNA. Positivity was significantly higher in patients older than 50 years and in the Gastroenterology department. Separately, 213 specimens with HBsAg S/CO values between 1.0 and 10.0 underwent HBV DNA confirmation testing. ROC analysis yielded an excellent area under the curve of 0.937, with an S/CO threshold of ≥3.04, achieving 100% sensitivity for HBV DNA detection. All specimens with S/CO values between 1.0 and 3.0 were HBV DNA-negative. In this study population, no serum HBV DNA positivity was detected among samples with S/CO values below 3.04, suggesting that HBV DNA testing may help avoid unnecessary confirmatory work-up in this range. Anti-HBc screening should be prioritized in older patients. S/CO-based confirmatory algorithm using HBV DNA testing may improve diagnostic accuracy and reduce unnecessary clinical interventions in routine hepatitis B screening programs.

## 1. Introduction

Hepatitis B virus (HBV) infection is a major global health concern, affecting approximately 290 million people worldwide, and can lead to severe liver diseases, including cirrhosis and hepatocellular carcinoma (HCC) [1]. The diagnosis of HBV infection is primarily based on serological tests and the detection of HBV DNA. Among the most commonly used biomarkers, Hepatitis B surface antigen (HBsAg) and Hepatitis B core antibody (anti-HBc) play critical roles in the diagnosis of acute and chronic infections. HBsAg is considered a marker of active infection, whereas anti-HBc can indicate either past exposure or active infection [2,3].

Turkey is generally classified as a region of intermediate HBV endemicity, with population-based HBsAg seroprevalence estimates typically reported in the range of approximately 2–8%, although considerable regional and population-based heterogeneity has been described across different provinces and healthcare settings [4,5].

Despite the availability of advanced screening technologies, the interpretation of serological markers often poses diagnostic dilemmas. The primary screening tool for HBV is the HBsAg, typically measured using chemiluminescent immunoassay (CLIA) platforms that provide a signal-to-cut-off (S/CO) ratio [6]. While high S/CO values are strongly indicative of infection, low-positive HBsAg S/CO values (often defined as 1.0 < S/CO < 10.0) are frequently associated with false positivity due to cross-reactivity, specimen interference, or the tail end of recent vaccination. The use of a confirmatory HBsAg assay aids clinicians in differentiating weakly reactive results from false-positive results. Therefore, it is crucial to determine the reactive S/CO values that should trigger confirmatory HBsAg assay [7,8,9,10].

Hepatitis B core antibody (anti-HBc) is the key serologic marker used to document a history of past HBV exposure. It is the first antibody to appear after infection, initially in the IgM form during the acute phase, before being progressively replaced by IgG anti-HBc, which generally remains detectable for life once approximately six months have elapsed since exposure. IgM anti-HBc therefore denotes recent/acute infection, whereas IgG anti-HBc denotes infection acquired in the more distant past. When IgG anti-HBc is detected in the absence of both HBsAg and anti-HBs, it is referred to as isolated anti-HBc. Isolated anti-HBc result can be interpreted in one of two ways: as a resolved infection in which protective anti-HBs titers have declined below the assay detection limit, or as evidence of occult hepatitis B virus infection (OBI), in which HBV DNA persists in the hepatic tissue despite an undetectable serum HBsAg level. OBI may occur together with or in the complete absence of anti-HBc and/or anti-HBs [11,12,13].

**Figure 1:**
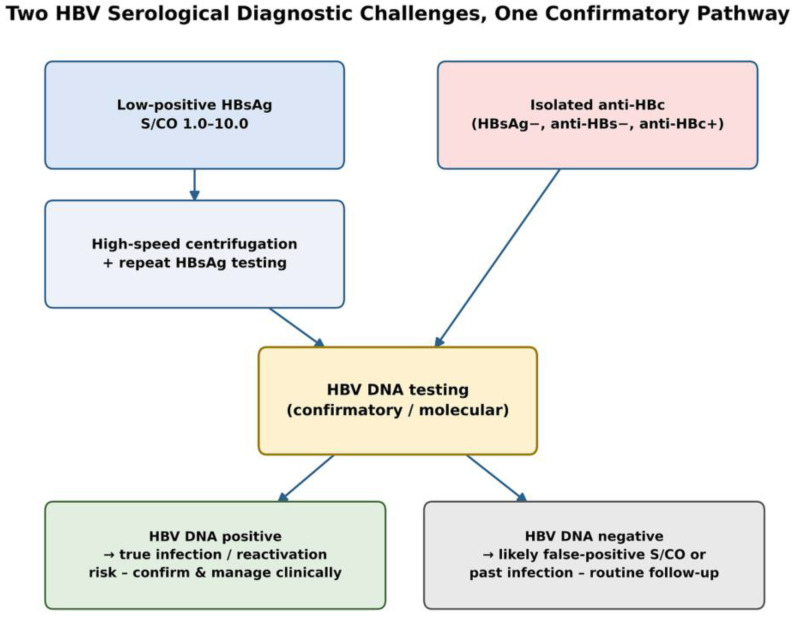
Diagnostic pathway integrating low-positive HBsAg S/CO values and isolated anti-HBc positivity through a common HBV DNA confirmatory step.

Low-level HBsAg S/CO positivity and isolated anti-HBc positivity represent the two points in HBV serology where interpretation is most often challenging. Accurate diagnosis is critical to avoid unnecessary psychological distress in patients and ensure appropriate clinical management. Although they differ in clinical practice, both are ultimately resolved in the same way: through HBV DNA confirmation [14,15,16]. Therefore, we analyzed these not as two unrelated tests, but as two applications of a single diagnostic principle: using HBV DNA testing to resolve serologically ambiguous HBV results, regardless of whether the ambiguity arises from a weak HBsAg signal or from an isolated anti-HBc profile (Figure 1).

This study aimed to evaluate the correlation between low-positive HBsAg S/CO values, isolated anti-HBc status, and HBV DNA levels to determine the necessity for molecular confirmation in these specific patient populations.

## 2. Materials and Methods

This retrospective study was conducted at the Karabuk Training and Research Hospital Medical Microbiology Laboratory using the results of HBsAg, anti-HBs, anti-HBc, and HBV DNA testing, along with demographic information of the patients between 2021 and 2025. In the serum samples sent to the microbiology laboratory, HBsAg, anti-HBs, anti-HBc, and anti-HBc IgM were detected using the chemiluminescence method with the ARCHITECT i2000 SR device (Abbott Diagnostics, Abbott Park, IL, USA). Serum specimens were isolated by centrifugation for 5 min at 2500 g and immediately tested for HBsAg. All specimens with an initial S/CO value of 1–10 were subjected to repeated HBsAg testing after high-speed centrifugation for 15 min. For HBV-DNA, viral nucleic acid extraction and purification were first performed using a Magnesia 16 automated nucleic acid extraction device (Anatolia Geneworks, Istanbul, Turkey). Quantitative HBV DNA tests were then performed on the extracted nucleic acids using qPCR with a Montania 4896 device (Anatolia Geneworks, Istanbul, Turkey) according to the manufacturer’s instructions. HBsAg, anti-HBs, anti-HBc total, and anti-HBc IgM were interpreted according to the manufacturer-defined reactive thresholds for reagent kits. Result interpretation was based on the following predefined thresholds: HBsAg, anti-HBc total, and anti-HBc IgM S/CO ratio ≥ 1.0 was reactive; anti-HBs: ≥10 mIU/mL was positive. For HBV-DNA quantification, viral nucleic acid extraction was performed using the automated Magnesia 16 Nucleic Acid Extraction System with the Magnesia Viral Nucleic Acid Extraction Kit (Anatolia Geneworks, Istanbul, Turkey), according to the manufacturer’s instructions. Quantitative HBV-DNA tests were performed using the qPCR method with a Montania 4896 device (Anatolia Genework, Istanbul, Turkey). The linear range of quantitation was 1 × 10^1^–1 × 10^9^ IU/mL and the analytic sensitivity was 10 IU/mL for Anatolia GeneWorks. Patient results were obtained from the hospital laboratory information system. Patients with missing data on HBsAg, anti-HBs, or anti-HBc were also excluded. The results of tests ordered by different physicians on different dates were not combined for the analysis. Each patient was included once in the study, and serum samples were analyzed based on the following inclusion criteria: HBsAg S/CO values between 1.0 and 10; an isolated anti-HBc-positive profile (defined as HBsAg-negative, anti-HBs < 10 mIU/mL, and anti-HBc IgG-positive), and the availability of HBV DNA test results, whether positive or negative. Conversely, patients with prior or ongoing treatment for HBV infection and those with incomplete or inconsistent serological test results were excluded from the study. HBV DNA testing was used as a targeted confirmatory test in patients with a low-positive HBsAg S/CO value or an isolated anti-HBc profile.

### Statistical Analysis

Statistical analyses were performed using SPSS version 22.0 software (SPSS Inc., Armonk, NY, USA). Descriptive statistics are presented as frequencies and percentages (n, %) for categorical variables. Pearson’s chi-square test was used to evaluate the distribution of anti-HBc positivity across demographic characteristics and in clinical departments. For analyses involving clinical departments with zero or low cell counts, Fisher’s exact test was applied. A multivariable binary logistic regression model was constructed to assess the independent predictors of isolated anti-HBc positivity and to control for potential confounding factors (age, sex, and clinical department). Adjusted Odds Ratios (aOR) and their corresponding 95% confidence intervals (CI) were calculated. Additionally, the Mann–Whitney U test was used to compare the distribution of continuous HBsAg S/CO values after high-speed centrifugation between the confirmed reactive and nonreactive groups. Receiver operating characteristic (ROC) curve analysis was performed to determine the optimal post-centrifugation HBsAg S/CO cutoff threshold for predicting HBV DNA positivity. Statistical significance was set at *p* < 0.05.

## 3. Results

Between 2021 and 2025, 17,356 patients were tested for anti-HBc, of which 3521 (20.3%) tested positive. By further evaluating the HBsAg and anti-HBs markers of the 3521 anti-HBc-positive cases, 506 (2.9%) cases that were HBsAg-negative, anti-HBs-negative, anti-HBc IgM-negative, and anti-HBc IgG-positive were defined as isolated anti-HBc cases. Furthermore, the HBV DNA test was positive in 16 (3.2%) patients. Some characteristics of patients with isolated anti-HBc positivity are shown in Table 1.

When the distribution of anti-HBc positivity was analyzed across clinical departments, the highest positivity rate was observed in the Gastroenterology outpatient clinic (5.09%), followed by Infectious Diseases (3.29%) and Internal Medicine (2.95%). The distribution of anti-HBc positivity rates across clinical departments was evaluated using Fisher’s exact test, and no statistically significant difference was observed (*p* = 0.344). The mean age of the 506 anti-HBc-positive patients was 63.87 ± 11.4 years (range, 23–93 years), with a median age of 65 years. The study population was stratified using an age threshold of 50 years. A significantly higher rate of anti-HBc positivity was observed in patients older than 50 years than in those aged 50 years or younger. Pearson’s Chi-square analysis demonstrated that this age-related distribution was highly significant (*p* < 0.001), indicating a notable effect or increased risk in the older population. No statistically significant relationship was observed between sex and anti-HBc positivity (*p* = 0.714). Multivariable logistic regression analysis was performed to control for potential confounding factors. The analysis confirmed that age > 50 years was a strong and independent predictor of isolated anti-HBc positivity, with patients > 50 years having a 3.81-fold higher odds of positivity compared to those aged 50 years or younger (aOR: 3.81, 95% CI: 2.92–4.97, *p* < 0.001). In contrast, the differences across clinical departments observed in the descriptive statistics did not retain statistical significance as independent predictors in the multivariable model once other confounders were adjusted. Sex also showed no independent association with isolated anti-HBc status.

Among the 506 patients with isolated anti-HBc positivity, 16 (3.2%) were positive for HBV DNA. Of these patients, 6 (37.5%) were referred by gastroenterology, 4 (25%) by internal medicine, 4 (25%) by infectious diseases, and 2 (12.5%) by rheumatology departments.

A total of 21,344 specimens were tested for HBsAg in our microbiology laboratory between 2021 and 2025. There were 228 specimens with initial S/CO values of 1–10; these specimens were subjected to high-speed centrifugation and repeated HBsAg testing. Of the 213 specimens with S/CO values of 1–10 after high-speed centrifugation, 35 (16.4%) were from patients in the confirmed reactive (HBV DNA-positive) group and 178 (83.6%) were from patients in the nonreactive (HBV DNA-negative) group. Patients with prior or ongoing antiviral treatment for HBV infection were identified through cross-referencing of the laboratory information system with the hospital’s system and outpatient clinical records for antiviral agents used in chronic HBV management; 43 patients were excluded. The baseline demographic and clinical department distributions of the 213 patients are presented in Table 2.

The overall mean age of the patients was 49.65 ± 19.42 years. The mean age was noticeably higher in the confirmed reactive group (55.63 ± 9.94 years) than in the nonreactive group (48.47 ± 20.59 years). Regarding age distribution, the majority of the confirmed reactive cases were clustered in the 41–60 age range (n = 22, 62.9%), and no pediatric cases (0–18 years) were detected within the confirmed reactive cases. In terms of sex distribution, females constituted a slight majority of the total study population (n = 114, 53.5%) and the confirmed reactive group (n = 20, 57.1%), whereas males accounted for 46.5% (n = 99) of the overall cases. The distribution of HBV DNA confirmation results across different HBsAg S/CO value ranges after high-speed centrifugation is presented in Table 3.

Specimens with S/CO values of 1.00–3.00 after high-speed centrifugation had 0% confirmed reactive results, whereas 61.4% were confirmed as reactive in the group with S/CO values of 5–10.

**Figure 2:**
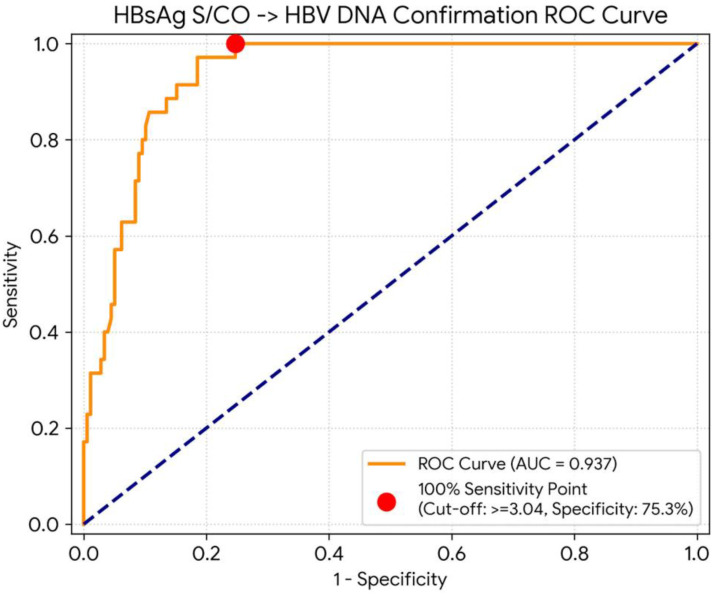
Receiver operating characteristic (ROC) curve for post-centrifugation HBsAg S/CO values in predicting HBV DNA positivity (AUC = 0.937, 95% CI: 0.898–0.977).

The median HBsAg S/CO value in the confirmed reactive group was 6.59, whereas it was 1.98 in the nonreactive group (*p* < 0.001). ROC analysis was performed for the S/CO values after high-speed centrifugation. The range of S/CO values that triggered mandatory confirmatory HBsAg testing was analyzed. Based on the results of our ROC analysis, HBsAg S/CO values ranging from 3.04 (100% sensitivity, 75.3% specificity) to 9.29 (17.1% sensitivity, 100% specificity) should trigger confirmatory HBV DNA testing. At the proposed cutoff of ≥3.04, the sensitivity of 100% (35/35) corresponded to a 95% confidence interval of 90.0–100%, and the specificity of 75.3% (134/178) corresponded to a 95% confidence interval of 68.3–81.4%. The area under the curve (AUC) was 93.7% (95% CI: 0.898–0.977), with a *p*-value of <0.001, demonstrating an excellent diagnostic performance in differentiating reactive cases from nonreactive ones (Figure 2).

## 4. Discussion

The interpretation of low-positive HBsAg results and isolated anti-HBc positivity presents a significant challenge in clinical practice. Although these two profiles arise from different mechanisms, in both scenarios, HBV DNA testing is the tool that distinguishes a clinically meaningful result from a serologically ambiguous one. Isolated anti-HBc positivity can also result from false positivity in assays used for anti-HBc screening, particularly among individuals from low-prevalence regions without risk factors for HBV infection [17,18]. Antibodies transferred through blood transfusions or from mother to baby can also cause isolated anti-HBc positivity. False HBsAg negativity is another potential reason. Additionally, false negativity can occur if the level of HBsAg is below the detection limit or if there is a mutation in the major antigenic determinant of the HBsAg marker. In a subset of these patients, isolated anti-HBc reflects OBI, with HBV DNA persisting in hepatic tissue despite HBsAg levels falling below the assay detection threshold. Although typically a minority, this subgroup carries a long-term risk of liver cirrhosis and hepatocellular carcinoma, and isolated anti-HBc positivity has also been associated with HBV reactivation in immunocompromised individuals [12,19,20,21]. Consequently, interpreting isolated anti-HBc reactive results is challenging [19,20,21]. The clinical significance of isolated anti-HBc positivity varies considerably depending on regional endemicity and the patient population. The global prevalence of isolated anti-HBc positivity varies from 0.1% to 20%. The rates of HBV DNA detected in individuals with isolated anti-HBc positivity vary between 0% and 30.5% across various studies [11,18,20]. In our study, the prevalence of isolated anti-HBc positivity was 2.9%, with a subsequent HBV DNA detection rate of 3.2% within this study. Compared to studies reported from Turkey, our rates are lower than those reported in a comprehensive multi-year study by the Cerrahpaşa Medical Faculty. This study demonstrated an isolated anti-HBc prevalence of 4.24% and a higher OBI rate of 4.80% among 31,939 analyzed cases. This difference may be explained by the fact that Cerrahpaşa is a large tertiary hospital serving high-risk patients, whereas our study included a more general population [18]. In another study conducted in the Trakya region, Davarcı et al. [20] reported a noticeably higher isolated anti-HBc prevalence of 6.0% over an extensive 8.5-year period, with an underlying HBV DNA (OBI) positivity rate of 2.9% within this study. Similar to our study, older patients and gastroenterology patients were found to have higher isolated anti-HBc positivity rates in that study. Global data also support a strong relationship between age and mortality. In a study from the United States, Hyun et al. [12] screened 7157 Korean American adults and found a very high isolated anti-HBc rate (10.8%). Our rate (2.92%) was lower because our study included a general hospital population with a lower risk. However, the age-related changes were very similar in both studies.

Reactivation of hepatitis B virus is a potentially life-threatening complication that primarily affects immunocompromised patients. It is not limited to HBsAg-positive individuals; HBsAg-negative, anti-HBc-positive patients also remain at risk, since HBV DNA can persist within hepatic tissue despite serum viral levels falling below the limit of detection. Among patients with isolated anti-HBc positivity, reactivation typically manifests as the reappearance of HBsAg and/or the emergence of detectable HBV DNA in serum [22,23,24,25]. Cancer incidence rises substantially with age. Omitting anti-HBc testing from routine screening may therefore leave older patients more vulnerable to undetected HBV exposure. In our study, isolated anti-HBc positivity was markedly higher in older age groups. This finding supports incorporating anti-HBc testing into routine screening protocols for this population [12].

Another important issue in hepatitis B serology is low HBsAg S/CO values. Weakly reactive HBsAg results, particularly those with low S/CO values, carry a considerable risk of false positives, which may lead to unnecessary patient anxiety, additional diagnostic workups, and inappropriate treatment decisions. High-speed centrifugation prior to retesting has been shown to reduce the interference of lipemia, fibrin, and other particulate matter, thereby improving the reliability of borderline results [16,26,27]. In our study, all specimens with S/CO values between 1.0 and 10.0 were subjected to high-speed centrifugation, followed by HBV DNA confirmation testing, which served as a reference standard. ROC analysis demonstrated that an S/CO threshold of ≥3.04 achieved 100% sensitivity, ensuring that no HBV DNA-positive specimens were missed. Pasaribu et al. [16] identified an S/CO range of 0.98–9.32 as the threshold requiring confirmatory HBsAg testing using the same CMIA method, with an AUC of 83.3%. The slightly higher AUC observed in our study (93.7%) may reflect differences in the patient population, sample composition, or the use of HBV DNA as a direct confirmatory measure rather than a neutralization-based assay. Wongchampa et al. [28] conducted a large-scale study of 72,496 specimens using the Elecsys HBsAg II kit and found that a cut-off index (COI) of 3.5 provided the best diagnostic performance, with 94.4% specificity and 88.3% sensitivity, and an AUC of 0.9429. The authors further noted that a S/CO threshold of ≥4.0 may be more practical in resource-limited settings, while a S/CO threshold of 13.0 achieved 100% specificity, recommending that each laboratory select a threshold suited to its clinical context. Purnamawaty et al. [29] reported that a COI of 1.08 was the optimal threshold for triggering confirmation using the CMIA method, achieving a specificity and sensitivity of 89.7% and 64.7%, respectively.

This study had several limitations. Firstly, it had a retrospective, single-center design based on data from a single tertiary hospital laboratory in Karabuk, which may limit the generalizability of the findings to other populations, referral patterns, or geographic regions with different HBV endemicities. All serological and molecular assays (HBsAg, anti-HBs, anti-HBc, and HBV DNA) were performed on a single diagnostic platform (Abbott ARCHITECT for serology and Anatolia Geneworks qPCR systems for HBV DNA); the results were not cross-validated on an alternative analyzer or assay platform. The number of HBV DNA-positive (confirmed reactive) cases in the low-positive HBsAg S/CO group was relatively small (n = 35 of 213 specimens), and the isolated anti-HBc group included only 16 HBV DNA-positive cases; these limited numbers reduced the statistical precision. Finally, the proposed S/CO threshold of ≥3.04 has not undergone external validation in an independent study or at another institution; therefore, it should be regarded as a preliminary, laboratory-specific proposal rather than a generalizable diagnostic threshold, pending confirmation in future multicenter or external validation studies. HBV DNA positivity was used as the reference standard for both the ROC-derived S/CO cutoff and the isolated anti-HBc analysis; however, HBV DNA and HBsAg are distinct biological markers of infection. A confirmatory HBsAg assay (e.g., a neutralization-based test) would have been a more appropriate reference standard for validation; however, such an assay was not available in our laboratory. The study population was restricted to patients for whom an HBV DNA result was available, which introduced a potential selection bias, since HBV DNA testing is preferentially ordered in patients with a higher pre-test probability of HBV infection. Obstetrics/Gynecology & Maternal Care subgroup in Table 2 comprised only 18 cases, of which a single case was HBV DNA-positive. This sample size is too small to support specific conclusions regarding HBV serological interpretation in pregnancy screening or the prevention of mother-to-child transmission, and the corresponding results should be regarded as suggestive only. Nonetheless, accurate determination of HBV infection status and viral replication level in pregnant women remains clinically important, as maternal viral load has been linked to the risk of intrauterine and perinatal transmission [30,31,32].

In conclusion, this study evaluated two clinically important but often under-recognized diagnostic challenges in hepatitis B serology: isolated anti-HBc positivity and low HBsAg S/CO values. In our study, an HBsAg S/CO threshold of ≥3.04 after high-speed centrifugation was associated with HBV DNA positivity; however, given the retrospective, single-center, single-platform design of this study and the limited number of HBV DNA-positive cases, this value should be regarded as a local, laboratory-specific candidate threshold rather than a validated and generalizable diagnostic cutoff. Its adoption in other settings would require confirmation through external validation in other studies. Incorporating HBV DNA testing into locally validated confirmatory algorithms for low-positive HBsAg S/CO values and extending anti-HBc screening, especially in older patients, has the potential to improve diagnostic accuracy, reduce unnecessary interventions, and support more cost-effective management of hepatitis B in routine clinical practices. We propose that laboratory-specific S/CO thresholds be validated at each institution, given that these values may vary depending on the analyzer platform, reagent lot, patient population, and local HBV endemicity.

## Figures and Tables

**Table 1:** Distribution of isolated Anti-HBc Positivity by Demographic Variables and Clinical Departments (2021–2025).

Variables	Total Tested n (%)	Anti-HBc Positivity, n (%)	*p*-Value
**Gender**			0.714
Male	7126 (41.06)	201 (2.82)	
Female	10,230 (58.94)	305 (2.98)	
**Age Group**			<0.001
≤50	6205 (35.75)	66 (1.06)	
>50	11,151 (64.25)	440 (3.95)	
**Department/Clinic**			0.344 ^a^
Gastroenterology	491 (2.83)	25 (5.09)	
Infectious Diseases	2736 (15.76)	90 (3.29)	
Internal Medicine	2747 (15.83)	81 (2.95)	
Rheumatology	10198 (58.76)	289 (2.83)	
Neurosurgery	466 (2.68)	10 (2.15)	
Dermatology	479 (2.76)	9 (1.88)	
Family Medicine	147 (0.85)	2 (1.36)	
Pediatrics	92 (0.53)	-	
**Total**	**17.356 (100%)**	**506 (2.92%)**	

^a^ Calculated using Fisher’s exact test due to the presence of zero-count cells.

**Table 2:** Demographic and Clinical Characteristics of Study Patients based on HBV DNA Confirmation Results.

Variable	Confirmed Reactive (n = 35)	Nonreactive (n = 178)	Total (n = 213)
**Age range n (%)**	55.63 (9.94)	48.47 (20.59)	49.65 (19.42)
0–18	0	11	11
19–40	1	51	52
41–60	22	54	76
>60	12	62	74
**Sex n (%)**			
Male	15 (42.9)	84 (47.2)	99 (46.5)
Female	20 (57.1)	94 (52.8)	114 (53.5)
**Clinical Department n (%)**			
Infectious Diseases	15 (42.9)	27 (15.2)	42 (19.7)
Internal Medicine & Subspecialties	6 (17.1)	51 (28.7)	57 (26.8)
Emergency & Primary/Occupational Care	6 (17.1)	19 (10.7)	25 (11.7)
Surgical & Physical Specialties	4 (11.4)	43 (24.2%)	47 (22.1)
Intensive Care & Dialysis	2 (5.7)	18 (10.1%)	20 (9.4)
Obstetrics/Gynecology & Maternal Care	1 (2.9)	17 (9.6%)	18 (8.5)
Other/Outpatient	1 (2.9)	3 (1.7%)	4 (1.9)

**Table 3:** Distribution of virological confirmation results within low-positive HBsAg S/CO ranges.

HBsAg S/CO Range After High-Speed Centrifugation	Number of Samples (n)	HBV DNA Detected (n)	Non-Reactive
1.0–3.0	132	-	132
3.1–5.0	37	8	29
5.1–10.0	44	27	17
Total	213	35	178

## Data Availability

All data obtained and analyzed for this clinical study are available from the corresponding author upon reasonable request.
